# Feasibility assessment of an 8-week attention-based training programme in the management of chronic spontaneous urticaria

**DOI:** 10.1186/s40814-021-00841-z

**Published:** 2021-05-03

**Authors:** Ridge Katie, Conlon Niall, Hennessy Martina, J. Dunne Pádraic

**Affiliations:** 1 Department of Immunology, St. James’s Hospital, Dublin, Ireland; 2 School of Medicine, Trinity College Dublin, Dublin, Ireland; 3 Wellcome trust HRB Clinical Research Facility, St. James’s Hospital, Dublin, Ireland; 4 Centre of Positive Psychology and Health, Royal College of Surgeons Ireland, Dublin, Ireland

**Keywords:** Chronic spontaneous urticaria, Meditation, Attention-based training, Stress

## Abstract

**Background:**

Chronic spontaneous urticaria is a common disorder that is poorly understood and frequently misdiagnosed. Psychological difficulties are a significant factor in dermatological diseases and may also aggravate symptom burden. Mind-body interventions are used as a complementary approach to alleviate symptoms in chronic diseases and may represent a valuable non-pharmacological approach in CSU.

**Methods:**

We sought to develop and evaluate the feasibility of an 8-week attention-based training (ABT) programme, coupled to biofeedback technology for CSU. Through convergent interviews, we gathered information from individuals with urticaria about possible links between stress, mood and skin symptoms. Using these data, we recruited 12 participants to engage in an amended ABT programme for patients with CSU, comprising eight 90-min sessions held weekly. Participants completed psychometric measures and measures of urticaria symptomatology as assessed by the urticaria control test, prior to and after the intervention. Adherence to ABT practice was measured using individual inner balance devices which tracked heart rate variability. We completed qualitative interviews after the intervention to obtain feedback on participant experience of the programme.

**Results:**

Participants with CSU described how their psychological wellbeing can be linked to skin symptoms, poor sleep and difficulty concentrating. An amended ABT programme was found to be an acceptable component of care in the management of CSU. Retention of participants in the programme was challenging with 33% participants dropping out of the programme. For those who did complete the programme, three participants exceeded weekly practice at week 8. A decrease in severity of urticaria symptomatology as measured by the urticaria control test was observed upon completion of the intervention. The most commonly cited barrier to implementation of the programme was the time commitment required.

**Conclusions:**

Integrating an ABT programme into routine clinical care for CSU patients is feasible and was deemed acceptable and valuable by individuals who took part. Further formal evaluation of ABT for CSU including the analysis of biochemical parameters is required to determine its role in the management of this distressing condition.

**Trial registration:**

This trial is registered with ISRCTN with study ID ISRCTN13672947. Registration took place on 22/09/2020 (retrospectively registered).

## Key messages regarding feasibility

- What uncertainties existed regarding the feasibility?

The acceptability of this programme in terms of participant interest was unclear. In addition, the opinion of patients with chronic spontaneous urticaria and angioedema as to whether their symptoms were affected by mood and psychological wellbeing was poorly understood.

- What are the key feasibility findings?

This programme was deemed beneficial by participants; however, retention over the course of the intervention was a challenge. The course was shortened due to the Covid-19 pandemic; however, despite this, participants demonstrated adherence to the skills taught and deemed them helpful.

- What are the implications of the feasibility findings for the design of the main study?

Integration of this programme into clinical care for chronic spontaneous urticaria and angioedema is feasible. The programme may be shortened in future formats to improve retention of participants. Comparing ABT with usual care and a non-ABT-based intervention will provide further information on the active ingredients of non-pharmacological approaches to managing this disease.

## Introduction

Chronic spontaneous urticaria (CSU) is a skin condition characterised by recurring episodes of wheals, angioedema or both lasting longer than 6 weeks [1]. CSU can also be referred to as chronic idiopathic urticaria and does not pertain to individuals with non-idiopathic urticaria, for example, physical urticaria [1]. CSU is a disorder that can be difficult to treat with up to 40% of patients having disease that is refractory to standard treatments [2]. Patients often present with significant psychological difficulties accompanying their disease, and this finding is reflected in the literature [1, 3–5]. Individuals with CSU have been shown to have significantly higher levels of self-reported depression and anxiety when compared to individuals without the disease [6]. In addition, impaired quality of life in CSU has been found to correlate with disease activity [7]. Itch is a bothersome symptom which typifies CSU, and problems with reduced productivity and absenteeism at work as well as sleep interference are common [8, 9]. It remains unclear as to whether psychological distress is a consequence of the disease or whether there is a link between psychological morbidity and the development of urticaria.

Mind-body interventions are not currently considered part of conventional treatments for CSU but have been combined with mainstream therapies in other dermatological diseases as part of a complementary approach. In conditions such as psoriasis, the role of stress and mood as a trigger of symptoms is long recognised, and emerging interventions examine these associations [10]. There is some evidence that meditation can lead to improved clinical outcomes in patients with psoriasis either used in isolation or when combined with traditional treatments [6, 9, 11]. Similarly, in atopic dermatitis, psychological approaches such as relaxation training and stress management have been used to reduce itch intensity, scratching and disease severity [8, 12].

Attention-based training (ABT) is based on the practice of mantra meditation and can be taught within a short timeframe [13]. Most recently, it has successfully been used as a non-pharmacological intervention to reduce burnout and improve sleep, immune regulation and heart rate variability in Emergency Department staff [13]. Mantra meditation, which is part of an ABT approach, has also been found to be of benefit in non-clinical populations [14].

### Objectives

This study sought to examine whether delivering a course in attention-based training to individuals with CSU would be deemed acceptable and beneficial by participants.

## Methods

### Design

This prospective single-site study sought to assess the feasibility and acceptability of an 8-week ABT programme for patients with CSU. The programme was completed in 2020 in a large teaching hospital in the Republic of Ireland. Recruitment took place between October and December 2019. The programme ran from January to March 2020. The study was conducted in accordance with guidelines for non-pharmacological clinical trials by the Consolidated Standards of Reporting Trials (CONSORT) group [15]. Institutional ethics committee approval was in place from the SJH/AMNCH Research Ethics Committee Secretariat.

### Participants

Fifteen patients with urticaria were informed of the study. We recruited twelve adults with a diagnosis of chronic urticaria with or without angioedema who had symptoms on at least 1 day per week and an urticaria control test score of <12 indicative of poorly controlled symptoms [16]. We excluded patients who had started an anti-depressant medication in the past 3 months or who had a diagnosis of an Axis I mental health disorder. Participants who met the inclusion criteria were informed of the study by medical staff in routine outpatient clinic. Patients wishing to participate gave informed written consent. A target of 12 participants was agreed by the research team in advance as an acceptable number for the group format of this programme.

### Intervention: 8-week ABT programme

The subsequent convergent themes common to the majority of the interviewees were used to inform and amend the existing ABT programme manual. The goal was to address the specific issues experienced by CSU patients in a bespoke ABT programme.

Recruited participants were invited to attend 8 consecutive, weekly ABT sessions, lasting 1 h and 30 min, facilitated by a qualified psychologist trained in ABT facilitation. Participants were asked to begin practicing ABT twice daily for 2 min at each sitting, 7 days a week (28 min total for week one). Practice times were incrementally increased each week culminating in a recommendation to practice for 10 min, twice daily, 7 days a week (140 min total for week 8).

Participants were provided with individual biofeedback devices (Inner Balance by HeartMath UK) as an objective measure of practice adherence. The ABT programme used mantra meditation to help disengage from thoughts, memories, emotions and symptoms. Subsequent weekly themes included developing a sustainable new habit; cultivating acceptance and self-compassion; and managing distractions and self-judgement, core beliefs, negative automatic thinking, sleep hygiene, the importance of exercise and the managing of checking behaviours and intense symptoms.

### Outcomes

#### Practice adherence

Adherence to daily practice was measured using individual Inner Balance devices (HeartMath UK). Inner Balance devices measured the duration (minutes) and quality of daily practice (coherence of heart rate variability in beats per minute over time). Researchers had access to practice data via the EmWave software (HeartMath UK) from consenting participants. Individual practice adherence was openly discussed during each ABT session.

### Psychometric measures and urticaria symptomatology

Participants were invited to complete the Depression and Anxiety Stress Scale (DASS 21) [17] a quantitative measure which provides three scores assessing depression, anxiety and stress; the PERMA profiler [18] a measure of psychological wellbeing assessing positive emotion, engagement, relationship, meaning and accomplishment providing separate scores for each domain; and the Five Facets of Mindfulness Questionnaire (FFMQ) which provides scores for observing, describing, acting with awareness, non-judgemental attitudes and non-reactive responses [19]. The above measures were completed 1 week prior the intervention (time 1) and 1 week after the intervention (time 2). Participants also completed the Urticaria Control Test which is a self-report measure of urticaria symptomatology [16].

### Convergent interviewing protocol

Participants were invited for an initial interview to examine how patients felt about the current status of their disease and the impact of internal and external stressors on their symptoms. We used convergent interviewing which represents a qualitative research technique to identify key aspects of an unknown process [20] such as stressful triggers of CSU symptoms. Convergent interviewing involves initially unstructured interviews lasting 1 h in duration to explore the research problem. After each interview, the transcripts were analysed and classified according to emerging themes. These themes were then integrated into the next interview in order to converge on the evolving themes in the topic area [21]. New potentially converging themes are sense-checked with subsequent interviewees to confirm or reject them.

Seven patients diagnosed with CSU agreed to take part in one to one interviews with a qualified psychologist. Interviews were recorded and later transcribed; audio recordings were destroyed once the patients had approved their transcripts. A set of five questions was introduced at various stages during the first interview, based on themes noted in the current research literature [22, 23]. The five questions were as follows:

1. Do you believe stress causes your symptoms?
2. Are early life stressful incidents linked to your symptoms?
3. Have you noticed the re-triggering of symptoms later in life by a stressor?
4. Have you any diagnosed allergies?
5. Is sleep an issue for you?

### Trial feasibility

Enrolment logs were recorded for all participants who met eligibility criteria. We sought to recruit a minimum of ten participants to the programme. Practice adherence was measured as previously described.

### Post-intervention semi-structured qualitative interviews

As part of the evaluation of the ABT programme, a subset of three participants was randomly selected to complete a qualitative interview. This interview was conducted by an independent researcher and was designed to elicit barriers and facilitators to engaging with the 8-week ABT programme. Participants were also invited to comment on the use of technology in the programme and provide feedback on how the programme was run.

### Primary outcomes

- Identification of barriers to implementation of the intervention through convergent interviewing techniques
- Recruitment and retention of participants
- Adherence to daily practice using individual biofeedback tools
- Feasibility of collecting outcome assessment data
- Qualitative assessment of participant experiences of the programme after completion

### Secondary outcomes

- Changes from baseline in perceived individual symptoms using the urticaria control test (UCT), post 8-week ABT programme
- Changes from baseline in depression, anxiety and stress using the depression-anxiety-stress score (DASS), post 8-week ABT programme
- Changes from baseline in biopsychosocial health using the PERMA-profiler, post 8-week ABT programme
- Changes from baseline in traits (attention, observational skills, non-reactivity and non-judgemental skills) associated with sustained meditation practice using the five-facet mindfulness questionnaire (FFMQ), post 8-week ABT programme.

### Statistical analysis

Statistics were calculated using the GraphPad Prism software. Mean and standard deviation values are denoted as follows: (*x* = $\overline{x}$ [*y*]). Power calculations were not calculated for this feasibility study due to insufficient existing data.

## Results

### Baseline characteristics

Twelve participants initially consented to participate in the programme (9F:3M). The age of participants ranged from 27 to 73 years ($\overline{x}$ = 49, SD = 13.4). One patient had a diagnosis of cholinergic urticaria. All others had chronic spontaneous urticaria. Five participants were prescribed at least two anti-histamines, and two participants were in receipt of omalizumab. The mean baseline UCT score was 7 whereby a score of <12 indicates poorly controlled urticaria.

### Convergent interviews in CSU

Table 1 illustrates the most commonly expressed themes in convergent interviews with demonstrative quotes. The majority of participants (71.4%) felt that stress acted as a trigger for their urticaria. Participants voiced a frustration with standard medical treatments for urticaria (85.7%), and four respondents (57.1%) had engaged in alternative approaches such as yoga in an attempt to manage their symptoms. Forgetfulness and family issues with one or both parents (e.g. alcoholism) were noted for the majority of interviewees. Additionally, 57.1% of interviewees voiced issues with restlessness, poor focus, over-thinking and checking behaviour (e.g. repeated checking whether appliances were left on or not). Interestingly, all seven interviewees used the phrase, “You just have to get on with it”, in relation to tolerating their symptoms. This stoicism and general lack of self-compassion was apparent for all participants (Table 1). The ABT programme was subsequently amended with particular emphasis on cultivating focus, present moment awareness, and self-compassion and managing checking behaviour.

**Table 1:** Themes emerging from each round of convergent interviewing of 7 participants diagnosed with CSU.

Common themes emerging over 7 interviews	Affirmative response to questions (***n***, %)	Representative quotes
Do you believe stress causes your symptoms?	5, 71.4%	“My urticaria is really stress-induced, a lot. Mostly stress induced or if I over-exert myself, like exercise too much.”
Early life stressful incident linked to symptoms	5, 71.4%	“Very notable in my teens. I grew up in a family with alcoholism and Alzheimer’s under the one roof which is not easy. Plus bullying at school.”
Re-triggering of symptoms later in life by a stressor	5, 71.4%	“It came again later with stress in college; house issues; partner split.”
Diagnosed allergies present including asthma	6, 85.7%	
Is sleep an issue?	6, 85.7	
**Themes emerging from first round interview**		
Frustration with medical treatment	6, 85.7%	“I suppose the length of time before I got to come in and see anybody, that doesn’t help either.” “It plays on your mind. Current medication Anti-histamines are not really working.”
Stoicism/lacking self-compassion	7, 100%	“You just have to get on with it; nobody will listen anyway”
Eldest in family	2, 28.6%	
Most responsible in family	6, 85.7%	
Strong internal critic “oughts and shoulds” statements predominate	3, 42.8%	
**Themes emerging from second round of interviews**		
Alternative therapy used	4, 57.1%	“I tried Yoga and Pilates and I can’t stand them because you have to be quiet and you don’t interact with people. I don’t like that.”
No strong support	3, 42.8%	“I don’t have the support that I really need.”
A worrier	2, 28.6%	
Forgetful	5, 72.4%	
Checking behaviour	4, 57.1%	“I regularly check things are turned off: light switches, cookers etc. It’s as if I’m on autopilot at times. If I’ve other things on my mind, I'm distracted.”
Exhausted much of the time	3, 42.8%	
Parent or family issues?	5, 71.4%	
**Themes emerging from third round of interviews**		
In your family, who gets the 2 am call (responsibility)?	3, 42.8%	“I usually get the call when things happen.”
Depression	2, 28.6%	
Forgetful of names?	3, 42.8%	
**Themes emerging from fourth round of interviews**		
Restlessness	4, 57.1%	“I can never relax or sit down. It always has to be me doing something. Being bored is the worst. Hate that. Constantly thinking about the next day. What am I going to do tomorrow? I know that I’m going a million miles an hour all the time. I need to relax.”
Poor focus	4, 57.1%	
Overthinking	4, 57.1%	
Are you organised?	2, 28.6%	

### Trial feasibility: recruitment and retention

Out of the twelve participants who consented to participate in the ABT programme, eight participants completed the programme. We consider completing the programme as follows: engaged in 4 out of 6 sessions prior to premature ending of the programme due to the COVID-19 pandemic, with active daily adherence to recommended practice, as measured by Inner Balance devices (see Table 2 below). The most commonly cited reason for drop out was the time commitment required by the programme.

**Table 2:** Retention and attendance statistics. Participants were only available to attend 6 out of 8 sessions, due to restrictions as a result of the COVID-19 pandemic in March 2020

	Week 1								
Participant	1	2	3	4	5	6	7	8	%attendance
CU1	x	x	x	X	x	x			**100**
CU2	x	x	x	X	x	x			**100**
CU3	x	x	x	X	x	x			
CU4	x	Absent	x	Drop-out	Drop-out	Drop-out	Drop-out	Drop-out	**-**
CU5	x	x	x	Absent	x	x			**83**
CU6	x	x	Drop-out	Drop-out	Drop-out	Drop-out	Drop-out	Drop-out	
CU7	x	x	x	X	Ill	Ill			**67**
CU8	x	x	x	X	x	x			**100**
CU9	x	x	Drop-out	Drop-out	Drop-out	Drop-out	Drop-out	Drop-out	
CU10	Absent	x	x	Drop-out	Drop-out	Drop-out	Drop-out	Drop-out	
CU11	x	x	x	X	x	x			**100**
CU12	x	Absent	x	X	x	x			**83**

### Practice adherence

**Fig. 1:**
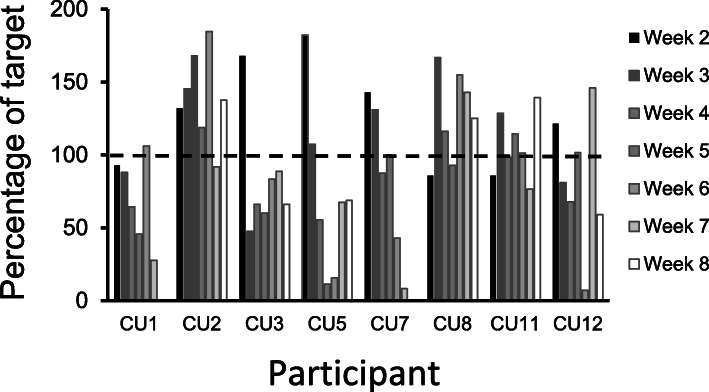
Adherence to weekly ABT practice. Adherence was measured using individual Inner Balance devices. Weekly practice targets over a 7-week period in minutes were 28, 42, 56, 70, 84, 98 and 112

Adherence values were calculated from weeks 2 to 8. Weekly practice targets over a 7-week period (in minutes) were 28, 42, 56, 70, 84, 98 and 112. Although weekly practice tapered towards the end of the programme, 42.8% participants exceeded weekly practice targets when measured at week 8 (Fig. 1). The combined average practice adherence values (percentage of target) over the course of the ABT programme for all participants were 61, 140, 83, 73, 73, 126, 106 and 83, respectively. Participant CU7 was ill and could not practice on weeks 5 and 6.

### Psychometric measures and urticaria symptomatology

Table 3 demonstrates baseline data including urticaria control test (UCT) scores, DASS 21, PERMA profiler and FFMQ scores for all participants at time 1. Time 2 data was returned for only seven participants. UCT scores indicative of urticaria symptomatology fell between time 1 and time 2. There was an improvement in positive psychology attributes and a reduction in stress, anxiety and depression between time 1 and time 2.

**Table 3:** Urticaria symptomatology and psychological analysis of participants using three validated survey instruments before (time point 1) and after (time point 2) the 8-week ABT programme

	Number of participants	Time Point 1 Mean (SD)	Time Point 2 Mean (SD)
Urticaria control test			
< 12 = poor control	4	6.7 (3.6)	12.3 (3.3)
DASS			
Depression	4	13.5 (11.9)	6 (6.2)
Anxiety	4	12.5 (2.3)	8 (3.4)
Stress	4	21.5 (12.3)	11 (4.7)
PERMA profiler			
Positive emotion	4	5.5 (2.1)	7 (1.3)
Engagement	4	9.2 (0.5)	7.3 (1.2)
Relationships	4	6.6 (1.7)	7.6
Meaning	4	5.7 (2)	7.7 (0.3)
Accomplishment	4	5.9 (2.2)	7.3 (1)
Overall wellbeing	4	6.5 (1.5)	7.4 (0.8)
Negative emotion	4	5.5. (3.1)	3.7 (0.6)
Health	4	3.8 (1.4)	4.7 (1.2)
Loneliness	4	3.5 (1.6)	5.5 (1.8)
FFMQ			
Observation skills	4	22.5 (2.7)	25.3 (2.5)
Describing skills	4	22.5 (3.4)	25.5 (6.1)
Awareness	4	24.3 (8.7)	27.8 (1.9)
Non-judgemental	4	23 (10)	29.8 (4)
Non-reactivity	4	15.8 (6.1)	24.3 (3.3)

### Post-intervention semi-structured qualitative interviews

Thematic analysis was used to identify key themes regarding barriers and facilitators to implementation (Table 4). Iterative discussion between two authors (KR and PD) refined analysis of themes. Summaries of findings were sent to the subset of participants for review; all affirmed the findings were accurate.

**Table 4:** Themes elicited in post-interview qualitative feedback

Benefits of participation in the ABT programme	“I found the therapy I would say very good. I seem to be able to control the flare ups…I find I’m not taking as much anti-histamines either” “I found that it pushed me to go back to doing twenty minutes (meditation) morning and evening and I have stuck to that”
Meeting others with the same condition	“It was nice to sit there and listen to other people’s stories. It just gives you a little bit of hope I think” “I was delighted to know that there was other people with similar conditions to what I have, because I thought that there wasn’t anyone”
Barriers to participating in ABT	“Just being too busy, not finding the time in the day” “Not managing to take the time in the morning and the evening, I’m not giving myself that time”
Facilitators to participating in ABT	“The gizmo that we had – it did kind of push you to do it because you know you’re being watched. It does make you more conscious of doing it” “I found the technology helpful to learn the technique of breathing”

## Discussion

The current study sought to design an attention-based training programme tailored towards patients with chronic spontaneous urticaria (CSU) and evaluate the feasibility of delivering this programme as part of clinical care. In order to create a programme that was relevant to this patient population, we used a convergent interview process to identify how individuals with urticaria understand links between their psychological wellbeing and skin symptoms. We sought to assess recruitment, retention and adherence of participants as well as changes from baseline in perceived individual symptoms of urticaria and psychological wellbeing prior to and post-intervention. We conducted qualitative interviews with a subset of participants to understand their experience of the programme.

### Themes emerging from convergent interviewing in CSU

There is growing evidence for the significant psychological burden of CSU, and this was confirmed by participants in the current study who felt that stress was an important factor in their disease. Participants voiced difficulties pertaining to poor concentration and sleep. Depression, anxiety, reduced quality of life and disturbed sleep are well documented in this cohort [3, 5, 7]. In addition, participants in the current study voiced a frustration with medical treatments for CSU and a desire to seek alternative therapies. The challenge of treating individuals with urticaria, particularly non-responders, is apparent and has led to a trend towards trialling alternative pharmacological and nonpharmacological therapies in CSU [24]. These findings highlight the importance of assessing the utility of a nonpharmacological approach that might address psychological and behavioural aspects of symptoms.

### Recruitment, retention and adherence

While recruitment to this study was relatively straightforward, retention of participants was a challenge. The Covid-19 pandemic impacted upon our intervention in its final weeks caused a reluctance to attend. The most common reason for withdrawal from the programme was the time commitment required. However, it is also a possibility that a meditation-based programme may not appeal to all individuals with CSU. Despite this, for individuals who persisted with the programme, all participants exceeded the weekly target of practice of ABT on at least one occasion.

### Perceived symptoms of urticaria and psychometric measures

We observed a reduction in perceived individual symptoms of urticaria upon completion of the ABT programme. Furthermore, measures of depression, anxiety and stress fell for all participants who returned follow-up data. While we are reluctant to over emphasise our findings given our small sample size, we remain confident that at a minimum did not have a negative impact upon participant wellbeing and is worth further examination with a larger sample. Data obtained can be used to develop power calculations and inform study size. In addition, qualitative interviews performed upon completion of the programme indicate that the programme was well liked and would be valued as a component of clinical care.

### Future development of the ABT programme

We have identified a number of solutions to try and facilitate participants of future programmes. We envisage that some sessions could be carried out using a virtual format. We also anticipate that when the programme is being run at a more significant scale, participants will have more options in terms of when they can attend the programme. The preliminary positive effects of our programme despite it having to be cut short by 2 weeks indicate that benefits from the programme might be achievable within a shorter time frame. Designing and evaluating a programme that is shorter in duration might be more attractive in terms of participant recruitment and retention.

## Conclusion

This feasibility study sought to assess whether an attention-based training programme could be successfully integrated into clinical care for patients with CSU. The programme was well tolerated with individuals reporting they felt they had benefited from taking part. In addition, we observed an improvement in psychological wellbeing and individual perception of urticaria symptoms in individuals who completed the programme. With only minor adjustment, we can assert that a randomised controlled trial to test the effectiveness of ABT as a component of CSU management will be feasible. Comparing ABT with usual care and a non-ABT-based intervention will provide further information on the active ingredients of non-pharmacological approaches to managing this disease.

## Data Availability

The datasets generated during the current study are/will be available upon request from the research team.
