# Correction to: Feasibility assessment of an 8-week attention-based training programme in the management of chronic spontaneous urticarial

**DOI:** 10.1186/s40814-021-00870-8

**Published:** 2021-06-29

**Authors:** Katie Ridge, Niall Conlon, Martina Hennessy, Pádraic J. Dunne

**Affiliations:** 1 Department of Immunology, St. James’s Hospital, Dublin, Ireland; 2 School of Medicine, Trinity College Dublin, Dublin, Ireland; 3 Wellcome trust HRB Clinical Research Facility, St. James’s Hospital, Dublin, Ireland; 4 Centre of Positive Psychology and Health, Royal College of Surgeons Ireland, Dublin, Ireland

**Correction to: Pilot Feasibility Stud 7, 103 (2021)**

**https://doi.org/10.1186/s40814-021-00841-z**

Following publication of the original article [1], the authors reported an error in presentation of the author names.

The correct presentation of the names are as follows:

1) Given name: Katie, Last name: Ridge

2) Given name: Niall, Last name: Conlon

3) Given name: Martina, Last name: Hennessy

4) Given name: Padraic, Given name: J, Last name: Dunne

The original article has been corrected.
